# Systemic Immune-Inflammation Index and Systemic Inflammatory Response Index as Predictors of Mortality in ST-Elevation Myocardial Infarction

**DOI:** 10.3390/jcm13051256

**Published:** 2024-02-22

**Authors:** Federica Marchi, Nataliya Pylypiv, Alessandra Parlanti, Simona Storti, Melania Gaggini, Umberto Paradossi, Sergio Berti, Cristina Vassalle

**Affiliations:** 1 Fondazione CNR-Regione Toscana Gabriele Monasterio, Ospedale G Pasquinucci, 54100 Massa, Italy; federica.marchi@ftgm.it (F.M.); alessandra.parlanti@ftgm.it (A.P.); simona.storti@ftgm.it (S.S.); umberto.paradossi@ftgm.it (U.P.); sergio.berti@ftgm.it (S.B.); 2 Institute of Clinical Physiology, National Research Council, Via G. Moruzzi 1, 56124 Pisa, Italy; melania.gaggini@ifc.cnr.it; 3 Fondazione CNR-Regione Toscana G Monasterio, Via G. Moruzzi 1, 56124 Pisa, Italy

**Keywords:** ST-elevation myocardial infarction, systemic immune-inflammation index, systemic inflammatory response index, prognosis, inflammation, mortality, neutrophils, platelets, lymphocytes

## Abstract

(1) **Background:** The systemic inflammatory response index (SIRI; neutrophil count × monocyte/lymphocyte count), and the systemic immune-inflammation index (SII; platelet count × neutrophil count/lymphocyte count) are recently proposed biomarkers to assess the immune and inflammatory status. However, data on SIRI and SII are still relatively lacking and do not definitively and exhaustively define their role as predictors of an adverse prognosis in acute myocardial infarction (AMI). The aim of the present study was to evaluate SII and SIRI determinants as well as to assess SIRI and SII prognostic power in ST-elevation myocardial infarction (STEMI). (2) **Methods:** A total of 105 STEMI patients (74 males, 70 ± 11 years) were studied (median follow-up 54 ± 25 months, 24 deaths). (3) **Results:** The main determinants of SIRI and SII were creatinine and brain natriuretic peptide (BNP) (multivariate regression). Patients with higher SIRI (>75th percentile, 4.9) and SII (>75th percentile, 1257.5) had lower survival rates than those in the low SIRI/SII group (Kaplan–Meier analysis). Univariate Cox regression revealed that high SIRI and SII were associated with mortality (HR: 2.6, 95% CI: 1.1–5.8, *p* < 0.05; 2.2, 1–4.9, *p* ≤ 0.05, respectively); however, these associations lost their significance after multivariate adjustment. (4) **Conclusions:** SIRI and SII association with mortality was significantly affected by confounding factors in our population, especially creatinine and BNP, which are associated with both the inflammatory indices and the outcome.

## 1. Introduction

Inflammation has a major role in all stages of the atherosclerosis process, with many different mediators involved in the complex inflammatory response, identified as crucial adverse prognostic factors in stable coronary artery disease and ST-elevation myocardial infarction (STEMI) patients [1]. Among these, the inflammatory parameters derived by a simple complete blood count have attracted interest as very simple, easily available, and low-cost biomarkers. For example, during acute myocardial infarction, the different blood cell types follow a typical temporal trend. In fact, neutrophils generally increase early and peak within one to three days; then, monocytes and platelets increase, whereas the number of lymphocytes decreases. In particular, calculated biomarkers obtained as ratios of two different cell types derived by a simple routine blood count (e.g., neutrophil-to-lymphocyte ratio (NLR), platelet-to-lymphocyte ratio (PLR), and monocyte-to-lymphocyte ratio (MLR)), have been studied and identified as prognostic factors in patients with coronary artery disease [1,2]. Recently, two novel indexes have been proposed, the systemic immune-inflammation index (SII) and the systemic inflammatory response index (SIRI) as novel additive inflammation-related tools that are useful for simultaneously assessing the inflammatory and immune status [3,4]. SII and SIRI might be more efficacious because they include three inflammatory parameters compared to the other previous three indices (NLR, PLR, and MLR). Specifically, they both include in their calculation neutrophil and lymphocyte number, with platelets being the other biomarker included in SII (index calculated as neutrophils × platelets/lymphocytes), whereas, in SIRI, monocytes are included (index obtained by the calculation of the following formula: neutrophil count × monocytes/lymphocytes). Practically, SIRI is a composite index encompassing the two previously proposed inflammatory indices, namely MLR and NLR; thus, an elevated SIRI level indicates a significant pro-inflammatory reaction, mediated by monocytes and neutrophils, together with a reduced lymphocyte-mediated anti-inflammatory effect. Instead, the SII index, which contains neutrophil, lymphocyte, and platelet count in its calculation, encompasses the platelets (critical factors that facilitate cell recruitment at the lesion sites and release a number of inflammatory mediators, thus increasing the inflammatory milieu) to combine the predictive power of both NLR and PLR.

Accordingly, both indexes have been related to poor prognosis in several types of cancers but found to be good predictors of events in patients with cardiovascular disease [3,4,5,6,7]. Some very recent studies reported the relationship between SIRI and SII and adverse prognosis, generally evaluated as composite events, in the cardiovascular field. Specifically, in patients with the acute coronary syndrome (ACS) undergoing percutaneous coronary intervention (PCI), SIRI was found to be a strong and independent risk factor for major adverse cardiovascular events (MACEs; overall death, non-fatal myocardial infarction, non-fatal stroke, and unplanned repeat revascularization) [8]. In a similar population of ACS patients undergoing PCI, SIRI was confirmed as an independent determinant of MACE, which included all-cause mortality and re-hospitalization for severe heart failure, during the follow-up period (median follow-up duration was 1142 days) [2]. In AMI patients, multivariable Cox regression models evidenced the association between SIRI and short-term mortality (30 and 90 days) [9]. Both SII and SIRI were found to also be able to predict the risk of in-hospital death in elderly AMI patients [10,11]. In a total of 5602 stable coronary disease patients who had undergone PCI, The SII score was found to be a better predictor for MACE (defined as nonfatal myocardial infarction, nonfatal stroke, and cardiac death, whereas secondary outcomes included a composite of MACE and hospitalization for congestive heart failure) when compared with traditional cardiovascular risk factors [6]. Moreover, the group of Su et al. reported the prognostic role of SII in determining the risk of short- and long-term mortality in ACS patients [12]. Additionally, in aortic stenosis patients who underwent transcatheter aortic valve implantation, an elevated pre-procedural SII has a predictive value for MACE (including stroke or transit ischemic attack, re-hospitalization, and all-cause short-term mortality) [13]. Another study, although showing the significance of SII as a predictor of length of hospital stay in ACS patients, unfortunately did not assess the relationship between SII and the risk of in-hospital mortality [14].

However, current studies on SIRI and SII and AMI are still relatively few, and have been performed in different patient populations and using different endpoints. Thus, the results are not definitive enough to draw definitive conclusions about their role as predictors of an adverse prognosis in this clinical setting. In particular, it is not still clear whether these hemochrome-related biomarkers related to inflammation have a direct and causal role, thus reflecting a real pathogenic mechanism for adverse prognosis, or simply represent biomarkers of an adverse risk profile linked to other more important prognostic risk determinants. This question is not purely formal or theoretical but substantial, because only by identifying SIRI and SII as a cause, rather than a consequence of other risk factors really related to cardiovascular adverse prognosis, may a targeted reduction in inflammatory mediators be more effective at improving outcomes. In this context, the relationship between SIRI and SII with other risk factors may disguise the link of these biomarkers to cardiovascular prognosis. 

Notably, SII and SIRI appear to be correlated to kidney function, as SII and SIRI values on admission were found to be independently associated with contrast-induced nephropathy development after PCI [15,16,17,18]. In fact, elevated SII and SIRI levels have been found to be also associated with kidney disease in T2D patients [19,20]. Interestingly, elevated SII levels were found to be strongly associated with the risk of heart failure (HF), where a significant negative correlation between the SII score and the left ventricular ejection fraction, cardiac output, and cardiac index was observed; these results suggest that this inflammation score not only indicates advanced disease but is also informative regarding the hemodynamic volume status [21,22]. SIRI was also found to be correlated with NTproBNP (which represents the N-terminal fragment of pro-brain natriuretic peptide (BNP), released by cardiomyocytes in response to different stimuli, including myocardial pressure, and cardiac volume overload), left ventricular remodeling, and systolic dysfunction in atrial fibrillation patients [23].

Despite SIRI and SII levels previously being found to be correlated with BNP/NTproBNP and creatinine, these variables are often not included as variables to adjust multivariate analysis (especially for natriuretic peptides) in the above-cited manuscripts examining the relationship between these indexes and adverse prognosis post-AMI. 

Thus, the aim of the present study was to evaluate the predictive value of SIRI and SII for mortality, as well as the SII and SIRI main determinants in the STEMI population.

## 2. Materials and Methods

Our Hub and Spoke network for STEMI in Northwestern Tuscany, Italy, began in April 2006 with the systematic use of Percutaneous Coronary Intervention (PCI—angioplasty with stent). The population of this 1658 km^2^ area is roughly 400,000. This program involved one Hub (Ospedale del Cuore di Massa) and five Spoke centers, one medical helicopter, and six advanced life support ambulances, with direct transmission of pre-hospital ECG to the catheterization laboratory (24 h/7 days PCI capability within 30 min of notification) activated by a single-call action. STEMI was defined according to ESC guidelines for STEMI criteria and management [24]. Patients underwent coronary angiography with subsequent percutaneous coronary intervention within 90 min of admission to the intensive care unit. 

Data from a total of 105 consecutive STEMI patients (74 men, age: 70 ± 11 years) admitted to the Coronary Care Unit of the Ospedale del Cuore G. Pasquinucci-Clinical Cardiology Department in Massa (Italy) were retrospectively analyzed in the study. Data from patients about demographic characteristics, cardiovascular history, cardiovascular risk factors, and instrumental and laboratory parameters were obtained by the hospital-computerized database [25]. 

Cardiovascular risk factors were defined as follows: obesity when body mass index >30 kg/m^2^; hypertension when blood pressure was more than 140/90 mmHg or the patient was currently using antihypertensive drugs; dyslipidemia when the patient was subjected to lipid-lowering treatments or presented fasting low-density lipoprotein levels of more than 150 mg/dL; type 2 diabetes (T2D) when the patient takes antidiabetic treatment; fasting glucose of more than 126 mg/dL (7 mmol/L) in two separate tests before the acute event; or there was a finding of HbA1c >6.49%. Smoking history was obtained by the clinical anamnesis and defined a current or former smoking habit. 

Left ventricular (LV) function was estimated by calculation of ejection fraction (EF) through echocardiographic 2-D measurement (modified Simpson’s rule with biplane planimetry); this parameter was defined as the ratio of the difference between the end-systolic and the end-diastolic volumes and the end-diastolic volume itself (%).

Inclusion criteria were as follows: (1) male and female adults; (2) age ≥ 50 years; (3) patients admitted to the Hospital Coronary Care Unit for chest pain and subsequently proven STEMI; (4) signed informed consent obtained from each patient (or from their relatives if the patient was unable to sign it). Exclusion criteria: (1) systemic diseases (e.g., systemic inflammatory diseases, cancer); (2) lack of a written informed consent. 

Blood samples were collected at admission in intensive care, immediately after performing PCI, and used for routine blood chemistry and blood count.

The SII and SIRI indexes were calculated using the following formula from the blood count performed at patient admission:

SII = platelet count × neutrophil count/lymphocyte count.

SIRI = neutrophil count × monocyte/lymphocyte count.

Standard medical therapy, including administration of aspirin, beta-blockers, ACE-inhibitors, diuretics, and anti-diabetic and lipid-lowering drugs, was given to eligible patients. Follow-up data and out-of-hospital cause of death were obtained from telephone interviews conducted by trained personnel or, in the case of unavailability of the patients, through medical electronic reports, hospital Discharge Records, and death certificates at the Municipal Civil Registries. The definition of cardiac death required the documentation of either significant arrhythmias, cardiac arrest, death attributable to congestive heart failure, or myocardial infarction in the absence of any other precipitating factor. 

## 3. Statistical Analysis

Continuous variables are expressed as mean ± SD, median (25–75th percentiles) for skewed variables, whereas categorical variables are reported as number (percentage). Owing to skewness, log transformations were used for some variables to perform statistical analysis; log-transformed values were back-transformed for data presentation. 

Statistical analysis included the Student’s t test for comparisons between continuous variables, and the χ^2^ test was used to assess differences between categorical variables. Regression analysis was performed to assess the relationship between continuous variables. Multiple regression analysis was applied to assess the effect of univariate significant variables in determining SII and SIRI levels. 

Percentiles were calculated from lowest to highest levels according to SII and SIRI values. Cumulative event rates were estimated by Kaplan–Meier survival curves and probability values were determined with the log-rank test. Statistical analysis also included Cox proportional hazard models to determine independent predictors of mortality. 

Model 1 reported the HR obtained at the univariate analysis for SIRI or SII; Model 2 was adjusted by adding age; Model 3 by adding sex; Model 4 by adding T2D; Model 5 by adding BNP; and Model 6 by adding creatinine to variables considered in each previous model.

The statistical package Statview, version 5.0.1 (SAS Institute, Abacus Concept, Inc., Berkeley, CA, USA), was used to perform analyses. A *p* value ≤ 0.05 was considered significant.

## 4. Results

Demographic and clinical characteristics of patients are summarized in Table 1. There were 47 (45%) patients with reduced left ventricular ejection fraction (LVEF < 50%), 59 (56%) with increased brain natriuretic peptide (BNP < 100 ng/L, hormone produced by cardiomyocytes in the heart ventricles in response to stretching caused by increased ventricular volume levels), and 15 (14%) with increased creatinine levels (>1.2 mg/dL, index of kidney function). In the whole population, mean (DS) levels of SIRI and SII correspond to 3.9 (3.4) and 1090 (905), respectively; moreover, a significant positive correlation was observed between SIRI and SII (r = 0.8, *p* < 0.001). As expected, female patients accounted for approximately a third of the overall population in agreement with previous results, as men are more likely to incur coronary heart disease [26].

### 4.1. SII Determinants in the STEMI Population

Of all variables reported in Table 1, a positive relationship was found between SII and BNP levels (r = 0.27, *p* < 0.01), and creatinine (r = 0.21, *p* < 0.05), in the whole population. A positive trend was also observed between SII and age, although without reaching statistical significance (r = 0.18, *p* = 0.07).

Data obtained by the multiple regression analysis are reported in Table 2, showing that BNP levels remained as independent determinant factors affecting SII in our population.

### 4.2. SIRI Determinants in the STEMI Population

A direct relationship was found between SIRI and age (r = 0.24, *p* < 0.05), BNP levels (r = 0.28, *p* < 0.01), and creatinine (r = 0.34, *p* < 0.01) in the whole population.

As reported in Table 3, after multiple regression analysis, creatinine levels remained as the only independent determinant factor affecting SIRI in our population.

### 4.3. Follow-Up

**Figure 1:**
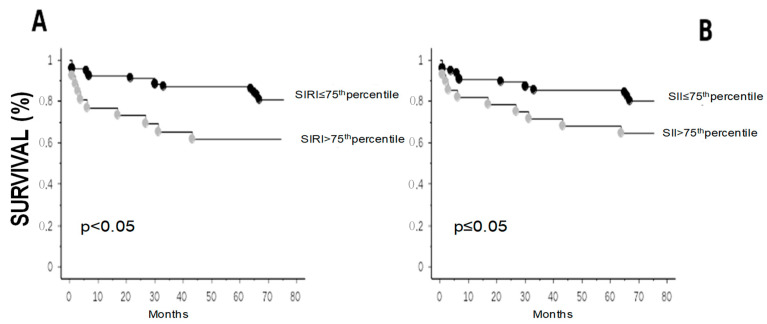
Kaplan–Meier survival curves for mortality according to SIRI (**A**) and SII levels (**B**).

During a mean follow-up period of 54 ± 25 months, 24 (22%) deaths were recorded. The Kaplan–Meier analysis showed that those patients in the high SIRI (>75th corresponding to 4.9) and SII (>75th corresponding to 1257.5) groups had lower survival rates than those in the low SII and SIRI groups (*p* < 0.05 and ≤0.05, respectively) (Figure 1, panel A and B, respectively).

**Figure 2:**
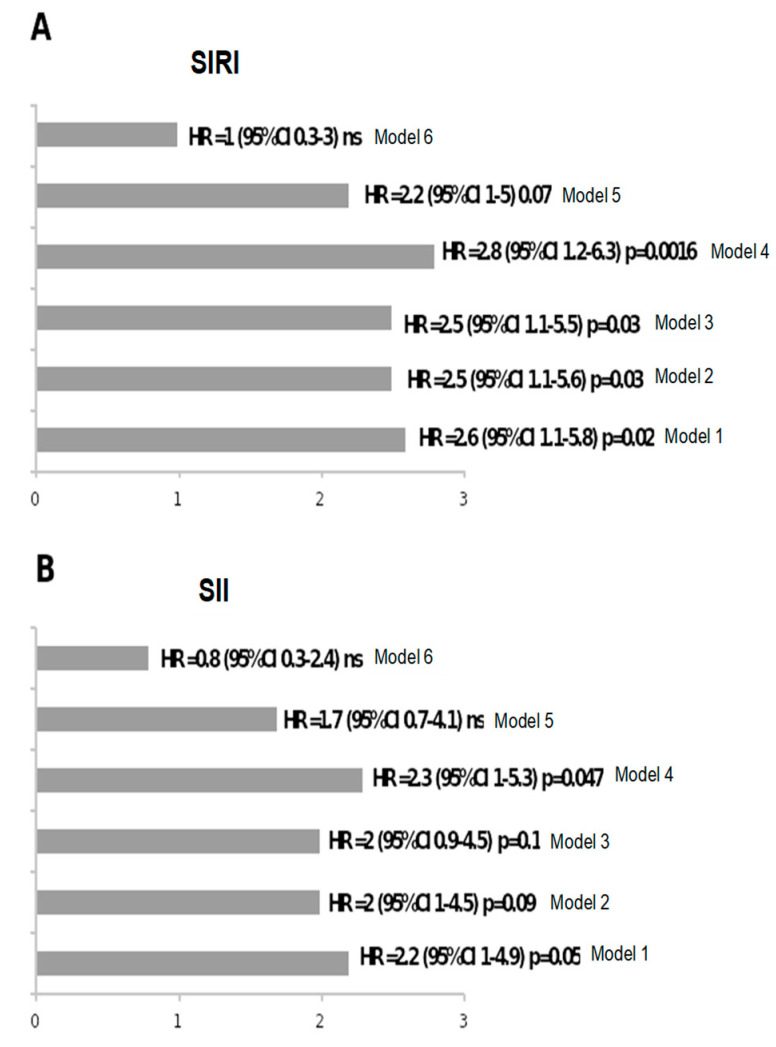
Hazard ratio (HR) of SIRI (**A**) and SII (**B**) for mortality according to univariate and multivariate Cox analysis (progressively adding new variables to those considered in the previous model) as follows: Model 1 reported the HR obtained at the univariate analysis for SIRI or SII; Model 2 was adjusted by adding age; Model 3 by adding sex; Model 4 by adding T2D; Model 5 by adding BNP; and Model 6 by adding creatinine.

According to the univariate Cox model, elevated SIRI and SII were associated with higher mortality, showing hazard ratio corresponding to 2.6 (95% confidence interval [CI]: 1.1–5.8, *p* < 0.05 and 2.2, 1.0–4.9, *p* ≤ 0.05), respectively. However, this significance was lost for SII with the fewest adjustments, and for both indices in the fully corrected model (Figure 2A,B).

## 5. Discussion

Our findings suggest that, although increased levels of SII and SIRI were associated with higher mortality in STEMI patients, these biomarkers lost their independent predictivity for mortality after adjustment for creatinine and BNP in the multivariate Cox analysis.

Atherosclerosis is generally defined as a chronic inflammatory disease of the vascular wall, and many data substantiate the importance of immune and inflammatory pathways in the progressive steps of vessel injury, until the precipitation of an acute coronary event such as AMI, which is an end-stage manifestation in patients with coronary heart disease. In particular, neutrophils, monocytes, platelets, and lymphocytes are all directly or indirectly involved in the onset and development of the atherosclerotic lesion, until the plaque rupture, a fact that provides a pathophysiological rationale for the significance of SII and SIRI in the atherosclerosis-related events and outcome. 

Thus, inflammatory-related biomarkers retain an important role in the development of cardiovascular disease, whereas a reduced inflammatory status decelerates the development of atherosclerotic injury and decreases the occurrence of cardiovascular events; accordingly, anti-inflammatory therapies have been found to be effective at lowering the incidence of cardiovascular events [27]. In this context, different inflammatory biomarkers (e.g., C reactive protein, NLR, PLR, and MLR) have been found to be related to short- and long-term adverse prognosis in AMI [28]. 

An acute ST-elevation myocardial infarction (STEMI), one of the most prevalent types of myocardial infarction, is an episode characterized by transmural myocardial ischemia resulting in myocardial injury or necrosis. In recent years, STEMI incidence and mortality have been increasing year by year.

Thus, further identification of inflammatory biomarkers that may better reflect the inflammatory burden and predict adverse clinical events is extremely important to reduce the mortality of STEMI patients, by applying appropriate targeted intervention measures to provide better patient quality of life.

The systemic immune inflammation index (SII) and system inflammation response index (SIRI) are two recently proposed inflammatory biomarkers that appear to be more comprehensive than other commonly used inflammatory indexes (e.g., NLR, PLR, and MLR) because they both integrate three inflammatory cells, thereby better reflecting the inflammatory and immune system status of patients compared to the other inflammatory indices that combine two cell types [3,4]. In fact, all the cells included in these biomarker calculations are key factors involved in the early and subsequent steps of the atherosclerotic injury development. Monocytes together with macrophages are immune cells that were first related to the development of atherosclerotic plaque, through recruitment of monocytes at the intima level and infiltration of monocytes that differentiate into macrophages; this drives inflammatory processes and foam cell production, and contributes to all stages that characterize atherosclerotic development [10]. Inflammation may drive abnormal increase in platelets, platelet activation, and increased adhesion to the surface of the endothelial cells, thus leading to local ischemic events, hypoxia, thrombosis, and clinically adverse manifestations such as myocardial infarction, stroke, and peripheral vascular diseases [29]. Neutrophils can also activate and enhance inflammatory responses, and induce plaque development, destabilization, and thrombosis [29]. Lymphocytes, in the form of B and T cells, B lymphocytes, and Th2 cells, have atheroprotective effects, whereas especially Th1 cells, induced by IL-12 and IL-18, enhance atherosclerosis damage [30]. A fall in lymphocyte count may be caused by excessive apoptosis, which reduces the immune system capacity and immune dysfunction, and promotes endothelial dysfunction and abnormal aggregation and thrombosis after platelet activation [31]. 

Although there are still few studies focusing on the relationship between SIRI and SII with cardiovascular prognosis, SII and SIRI have been more extensively studied in the cancer fields, where their levels have been found to be associated with tumor progression and poor survival outcomes in different types of malignancies, suggesting that that these biomarkers may be a prognostic indicator in various malignant conditions; moreover, currently these biomarkers are used as biomarkers for the assessment of the inflammation burden in different pathophysiological conditions [32,33,34].

Accordingly, although SII is more studied, both parameters have shown promise as tools for cardiovascular risk stratification and as good predictors of composite endpoints for adverse short- and long-term occurrence of cardiovascular events [1,6].

In this context, SIRI seems to have a stronger association with outcome events than SII, a fact that may be due to the different components used in the calculation of the two indices, as the mechanisms induced by platelet and monocyte in plaque instability may be different [1]. 

Nonetheless, evidence obtained in the general population showed an association between SII and the risk of future stroke, but not for coronary artery disease or AMI. In particular, data from a Chinese population indicated that an elevated SII is related to the incidence of cerebrovascular diseases, but not to ischemic cardiovascular disease or ACS [35].

These results thus represent the risk condition of people living in the northern part of China. The NHANES database represents a nation-wide representative cohort with different ethnicities, thus providing more generalized results. Accordingly, data obtained in a general population older than 60 years, including 42,875 subjects from the NHANES database, evidenced that both indices are significantly related to cardiovascular death and all-cause death [36]. In a ten-year follow-up study including 85,154 subjects, higher rates of stroke and all-cause death were observed in correspondence to high levels of SII and SIRI, while the high risk of MI was only independently related to high SIRI [37]. However, despite the adjustment for several relevant confounders, additional unmeasured factors may exist, which may cause spurious associations. In particular, different studies showed no adjustment for creatinine and/or natriuretic peptides, which are key prognostic factors for mortality in AMI patients and, as shown by our results, significant independent determinants for SIRI and SII, respectively [12,37]. Accordingly, both elevated SII and SIRI are associated with contrast-induced nephropathy and impairment of kidney function [15,16,38]. BNP is an index of cardiac function and a sensitive and specific biomarker for assessing the extension of myocardial infarction [39]. Its prohormone, the N-terminal fragment of the brain-type natriuretic peptide (NT-proBNP), has been found to be correlated to SII [40]. Moreover, SII and NT-proBNP represented independent determinants of clinical prognosis in acute STEMI patients when considering a composite endpoint (including all-cause mortality, re-hospitalization, nonfatal myocardial infarction, acute heart failure, and non-fatal stroke), and they were positively correlated [41]. Thus, confounding variables (e.g., creatinine and BNP levels) may alter the association of inflammatory parameters with the outcome in question and should always be considered in the statistical analysis.

Interestingly, we found a significant correlation between SII and logFibrinogen (r = 0.23, *p* = 0.02) in a subgroup of patients (*n* = 101), which in turn was significantly correlated with logBNP (r = 0.5, *p* < 0.001). Fibrinogen is an acute-phase protein affecting blood clotting, and a recognized risk factor of cardiovascular disease, especially regarding AMI. Elevated blood fibrinogen levels are involved in cardiovascular events through fibrin production and accumulation, affecting the evolution of the atherosclerotic lesion and increased blood viscosity related to thrombus formation; thus, this fact provides biological plausibility for the relationships found [42]. Thus, the additive value of SII over fibrinogen in terms of prognosis must be evaluated in future.

### Limitations and Strengths of the Study

The present study has some limitations. First, this was a retrospective single-center study, with a relatively small sample size. Thus, a post hoc power analysis was performed using the program G*Power 3.1, choosing a medium effect size of 0.5. Taking into account this effect size, a study power (1 − β) of 0.99 was expected with an α value of 0.05. This study evaluated the inflammatory status of STEMI patients upon admission, whereas temporal variations in SII and SIRI levels could affect the outcome; this fact needs to be explored in future studies. Moreover, the population enrolled was limited to Italian subjects, and thus the conclusion requires further validation before being extended to other ethnic groups. Nonetheless, our analysis was adequately controlled for the potential key confounders. Notably, patients also completed a long-term follow-up. Further analyzes to verify the level of prediction of these biomarkers in subgroups of patients, such as in young AMI subjects or in patients with reinfarction, also remain to be evaluated in future studies.

## 6. Conclusions

Our results suggested that creatinine and BNP are associated with both the risk factors of interest (SII and SIRI) and the outcome, and as such reduce the power of prediction of SII and SIRI for mortality in STEMI patients. 

## Figures and Tables

**Table 1:** Demographic, clinical, instrumental, and laboratory characteristics of STEMI patients in the overall population.

Variable	Value
Number	105
Age (years)	70 ± 11
Males/Females	74 (70)/31 (30)
Diabetes	20 (19)
Hypertension	63 (60)
Dyslipidemia	52 (49)
BMI (Kg/m^2^)	27 ± 5
Smoking history (current or former smoking habit)	44 (43)
LVEF (%)	48 ± 9
BNP (ng/L)	128 (51–245)
Creatinine (mg/dL))	1 ± 0.3
Neutrophils (10^3^/µL)	6 (4.7–8.1)
Lymphocytes (10^3^/µL)	1.8 (1.4–2.1)
Monocytes (10^3^/µL)	0.8 (0.6–1)
Platelets (10^3^/µL)	234 ± 76

Data are reported as mean ± DS, median (25–75th percentiles), or number (%).

**Table 2:** Multiple regression between SII levels and different risk factors.

Variable	Standard Coefficient	t-Value	*p* Value
Creatinine	0.17	1.6	ns
BNP	0.26	2.5	<0.05

ns = not significant.

**Table 3:** Multiple regression between SIRI and different risk factors.

Variable	Standard Coefficient	t-Value	*p* Value
Creatinine	0.28	2.7	<0.01
BNP	0.15	1.3	ns
Age	0.1	0.8	ns

ns = not significant.

## Data Availability

Data are available for research purposes on request to authors.
